# Dental follicle stem cells rescue the regenerative capacity of inflamed rat dental pulp through a paracrine pathway

**DOI:** 10.1186/s13287-020-01841-1

**Published:** 2020-08-03

**Authors:** Hong Hong, Xiaochuan Chen, Kun Li, Nan Wang, Mengjie Li, Bo Yang, Xiaoqi Yu, Xi Wei

**Affiliations:** 1 Hospital of Stomatology, Guanghua School of Stomatology, Guangdong Provincial Key Laboratory of Stomatology, Sun Yat-sen University, Guangzhou, 510055 People’s Republic of China; 2 Department of Stomatology, The Third Affiliated Hospital of Sun Yat-sen University, Guangzhou, 510630 People’s Republic of China; 3 Key Laboratory of Green Chemistry and Technology, Ministry of Education, College of Chemistry, Sichuan University, Chengdu, 610064 People’s Republic of China

**Keywords:** Dental follicle stem cells, Immunomodulation, Paracrine effect, Pulpitis, Regenerative endodontics

## Abstract

**Background:**

Pulpitis is a common dental disease characterized by sustained inflammation and impaired pulp self-repair. Mesenchymal stem cell-based minimally invasive vital pulp therapy (MSC-miVPT) is a potential treatment method, but its application is limited by the difficulty in acquiring MSCs. We recently revealed the immunomodulatory effects of rat dental follicle stem cells (rDFSCs) on acute lung injury. The present study focused on the paracrine effects of rDFSCs on the inflammation and regeneration of rat injured dental pulp to detect whether DFSCs are a potential candidate for MSC-miVPT.

**Methods:**

Conditioned medium from rDFSCs (rDFSC-CM) was applied to lipopolysaccharide (LPS)-induced inflammatory rat dental pulp cells (rDPCs). The inflammation and regeneration of rDPCs were detected by RT-qPCR, Western blotting, immunofluorescence staining, Cell Counting Kit-8 (CCK-8) assay, flow cytometry, wound-healing assay, and Masson’s staining. The effects of rDFSC-CM on inflamed rat dental pulp were further evaluated by hematoxylin-eosin and immunohistochemical staining.

**Results:**

rDFSC-CM downregulated the ERK1/2 and NF-κB signaling pathways, which resulted in suppression of the expression of IL-1β, IL-6, and TNF-α and promotion of the expression of IL-4 and TGF-β, and these findings lead to the attenuation of rDPC inflammation. rDFSC-CM enhanced the in vitro proliferation, migration, and odontogenic differentiation of inflammatory rDPCs and their in vivo ectopic dentinogenesis. Furthermore, rDFSC-CM inhibited inflammatory cell infiltration in rat pulpitis and triggered Runx2 expression in some of the odontoblast-like cells surrounding the injured site, and these effects were conducive to the repair of inflamed dental pulp.

**Conclusions:**

rDFSC-CM exhibits therapeutic potential by rescuing the regeneration of the inflamed rat dental pulp through an immunomodulatory mechanism, indicating the application prospects of DFSCs in biological regenerative endodontics.

## Background

Dental pulp inflammation, which is also called pulpitis, is an inflammatory dental disease that causes the destruction of pulp tissues and eventually results in functional loss of the pulp. As bacteria and their toxic components penetrate into the pulp, inflammatory responses from resident cells, including dental pulp cells (DPCs), macrophages, and other immune cells, are initiated to eliminate invading microbes [1]. However, these antimicrobial activities allow the easy propagation of sustained inflammation, which is enormously destructive to the vital pulp and ultimately leads to tissue necrosis and prevention of innate self-repair of the pulp [2–4]. Hence, the attenuation of excessive inflammation and the preservation of pulp vitality have been the most substantial challenges in modern endodontics. True innovation in the development of novel minimally invasive biotherapies for vital pulp preservation is critically needed.

Mesenchymal stem cells (MSCs) affect neighboring immune cells through direct cell-to-cell contact and/or various paracrine factors, such as indoleamine 2,3-dioxygenase (IDO), prostaglandin E2 (PGE2), TGF-β, and hepatocyte growth factor (HGF) [5–8]. On the one hand, MSCs exert immunosuppressive effects by suppressing the secretion of proinflammatory factors while increasing the expression of anti-inflammatory mediators of immune cells [9, 10], and on the other hand, MSCs regulate immune responses by eliciting the reprogramming of macrophages, suppressing the activation of B and T cells, and facilitating the proliferation of Treg cells [6, 7, 11, 12]. Based on their immunomodulatory capacities, MSCs are prospective candidates for use in cell-based therapies for various immune and inflammatory diseases [13–15], but the application of these therapies is limited by difficulty in acquiring MSCs.

The development of therapeutic applications for dental MSCs offers significant potential. The opportunity to isolate these cells from extracted teeth provides a ready source of dental MSCs, which eliminates the difficulty associated with their acquisition. Our previous study [16] showed that conditioned medium (CM) from rat dental follicle stem cells (rDFSCs), which are a type of dental MSCs, can effectively alleviate acute lung injury by downregulating the expression of proinflammatory cytokines and reprogramming macrophages toward the anti-inflammatory M2 phenotype. Protein arrays have revealed that rDFSC-CM is rich in numerous bioactive factors. Herein, we hypothesized that rDFSC-CM is also a potential option for ameliorating pulp inflammation and facilitating tissue regeneration due to its immunomodulatory effects. In the present study, we investigated the immunomodulatory effects of rDFSC-CM on lipopolysaccharide (LPS)-induced inflammatory rat dental pulp cells (rDPCs) and pulpitis model rats. Furthermore, we explored the effects of rDFSC-CM on the regeneration capacity of inflammatory rDPCs in vitro and the self-repair of injured dental pulp tissue in vivo.

## Methods

### Preparation of rDFSC-CM

Sprague-Dawley (S-D) rats were obtained from the Laboratory Animal Center at Sun Yat-sen University, and dental follicle tissues were isolated at postnatal day 7, minced into small pieces, and digested in α-minimum essential medium (αMEM, Gibco, USA) supplemented with 3 mg/mL collagenase I and 4 mg/mL dispase II (Sigma-Aldrich, USA) at 37 °C for 30 min. The explants were then cultured in αMEM containing 20% fetal bovine serum (Gibco, USA), 100 U/mL penicillin, and 100 mg/mL streptomycin (Gibco, USA) at 37 °C in a humidified atmosphere with 5% CO_2_. Once they reached 90% confluence, the cells were passaged at a 1:3 ratio, and cells from passages 3 to 5 were used in the experiments. When cells reached 70% confluence, the CM was collected after a 24-h interval by exchanging the complete medium with fresh serum-free medium. The CM was centrifuged at 1000×*g* for 5 min and filtered through a 0.22-μm strainer, and the culture supernatant was then stored at − 80 °C. To prepare the rDFSC-CM, the obtained medium was diluted 50% with an equal volume of αMEM.

### Isolation and culture of rDPCs

For the isolation of rDPCs, 5-week-old S-D rats were obtained from the Laboratory Animal Center at Sun Yat-sen University. After intraperitoneal anesthesia with 10% chloral hydrate, the maxilla and mandible were separated, and the dental pulp tissues of the incisors were transferred to an 8-cm^2^ culture dish and washed with phosphate-buffered saline (PBS, Sigma-Aldrich, USA) containing 2% penicillin-streptomycin (Sigma-Aldrich, USA). The minced pulp tissue was digested with 3 mg/mL collagenase I and 4 mg/mL dispase II at 37 °C for 30 min. The cells were cultivated in αMEM containing 20% FBS and 2% penicillin-streptomycin in a T25 cell culture flask at 37 °C in an atmosphere with 5% CO_2_. Cells from passages 3 to 5 were used in the experiments.

### Immunofluorescence staining of vimentin and cytokeratin in rDFSCs and rDPCs

Immunofluorescence staining was performed according to standard protocols. In brief, the cells (2 × 10^3^ cells/well) were plated in 12-well plates (Corning, USA) and cultured for 24 h. The media were then removed, and the cells were fixed with 4% paraformaldehyde (Sigma-Aldrich, USA) for 15 min, permeabilized with 0.1% Triton X-100 (Sigma-Aldrich, USA) for 15 min, and incubated with 10% donkey serum for 30 min at room temperature. The plates were then incubated with anti-vimentin antibody (Abcam, USA) at 1:200 dilution and anti-cytokeratin-14 antibody (Affinity, China) at 1:100 dilution overnight at 4 °C. Alexa Fluor® 488 donkey anti-rabbit IgG and Alexa Fluor® 594 donkey anti-rabbit IgG (Life Technologies, USA; 1:400) were used as the secondary antibodies. The samples were scanned and photographed under a Panoramic MIDI slide scanner (3DHISTECH, Hungary).

### Flow cytometric analysis of surface markers of rDFSCs and rDPCs

The phenotype of rDFSCs and rDPCs was identified by flow cytometric analysis. The MSC phenotyping cocktail comprised both positive (CD29-FITC, CD44/CD90-PE, BD Bioscience, USA) and negative (CD34-PE, CD45-FITC, BD Bioscience, USA) fluorochrome-conjugated monoclonal antibodies. IgG1-FITC and IgG1-PE (BD Bioscience, USA) were used as isotype controls. Third-passage rDFSCs and rDPCs were suspended to 5 × 10^5^ cells/mL in PBS solution, stained with different antibodies for 30 min at 4 °C, washed with PBS, resuspended in FACS buffer, and analyzed using a MOFlo™ high-performance cell sorter (Beckman Coulter, USA).

### Evaluation of osteogenic and adipogenic capabilities of rDFSCs and rDPCs

The cells (2 × 10^5^ cells/well) were loaded in six-well plates (Corning, USA). Once the cells reached 80% confluence, the medium was changed to commercial osteogenic medium (Cyagen Biosciences, China) or adipogenic medium (Cyagen Biosciences, China). After 14 days of induction, the cells were fixed in 4% paraformaldehyde (Sigma-Aldrich, USA) for 30 min and then subjected to Alizarin Red staining (Cyagen Biosciences, China) to reveal calcium depositions or Oil Red O staining (Cyagen Biosciences, China) for the observation of lipid droplets. The cells were imaged with a Fluorescence Inversion Microscope System (Carl Zeiss, Germany).

### LPS-induced inflammatory rDPCs

The rDPCs (1 × 10^5^ cells/well) were seeded in six-well plates and cultivated in αMEM containing 10% FBS and 2% penicillin-streptomycin. When the cells reached 70% confluence, the culture medium was changed to αMEM with LPS (0.5 mg/L, *Escherichia coli*, Sigma-Aldrich, USA). Cells that were not stimulated with LPS were used as a control.

### Real-time quantitative PCR

The gene expression of inflammatory cytokines in rDPCs was investigated by real-time quantitative PCR using SYBR Green Mix (Roche, Germany). rDPCs were treated with αMEM or rDFSC-CM for 3, 6, and 9 h, and total RNA was isolated from the inflammatory rDPCs using TRIzol (Invitrogen, USA). First-stand cDNA was synthesized from 2 μg of total RNA. The PCR primers were synthesized by Invitrogen (Life Technologies, USA), and their sequences are listed in Table 1. The data were acquired and analyzed using a LightCycler 480 system.

**Table 1:** Primers for inflammatory cytokines used in the real-time quantitative PCR assay

Gene	Primer sequence	
*IL-1β*	Forward	CCCTGAACTCAACTGTGAAATAGCA
	Reverse	CCCAAGTCAAGGGCTTGGAA
*IL-6*	Forward	ATTGTATGAACAGCGATGATGCAC
	Reverse	CCAGGTAGAAACGGAACTCCAGA
*TNF-ɑ*	Forward	TCAGTTCCATGGCCCAGAC
	Reverse	GTTGTCTTTGAGATCCATGCCATT
*IL-4*	Forward	CAAGGAACACCACGGAGAAC
	Reverse	CTCAGTGAGTTCAGACCGCT
*TGF-β*	Forward	GAAGTCACCCGCGTGCTAATGG
	Reverse	GTGTGTCCAGGCTCCAAATGTAGG
*IL-10*	Forward	GTGTGTCCAGGCTCCAAATGTAGG
	Reverse	CAAGGCTTGGCAACCCAAGTA
*β-Actin*	Forward	CCTCTTTGCATGTCTCACTC
	Reverse	AATGTCACGCACGATTTCC

### Western blot analysis

The cells were cultured in αMEM or rDFSC-CM with 0.5 mg/L LPS for 0, 15, 30, 60, and 120 min, and protein was then collected by lysis with phosphatase inhibitor and protease inhibitor (Thermo Fisher Scientific, USA). The protein contents were quantified using a bicinchoninic acid protein assay kit (Biocolors, China). Thirty micrograms of proteins was separated on sodium dodecyl sulfate-polyacrylamide gels and transferred to nitrocellulose membranes (Millipore, USA). The membranes were probed with the following primary antibodies overnight at 4 °C: anti-p-p38 MAPK, anti-p38 MAPK, anti-p-ERK 1/2, anti-ERK1/2, anti-p-SAPK/JNK, anti-SAPK/JNK, anti-p-p65 NF-κB, anti-p65 NF-κB (1:1000, Cell Signaling Technology, USA), and anti-vinculin (1:5000, Abcam, USA). The membranes were subsequently washed for 30 min and incubated with horseradish peroxidase-conjugated secondary antibody (Cell Signaling Technology, USA) at 1:5000 dilution. The immunoreactive proteins were visualized by enhanced chemiluminescence (ECL; Millipore, USA), and the densities of the protein bands were quantified using ImageJ version 1.50i software (Bethesda, USA).

### Immunofluorescence analysis

The subcellular location of NF-κB p65 in inflammatory rDPCs after treated with αMEM or rDFSC-CM for 60 min was examined by immunofluorescence staining. Briefly, rDPCs were fixed in 4% formaldehyde solution for 15 min, permeabilized with 0.1% Triton X-100 for 15 min, and incubated with 10% swine serum (ImmunoReagents, USA) for 1 h at room temperature. The samples were stained with anti-NF-κB p65 antibody (Cell Signaling Technology, USA) at 1:400 dilution overnight at 4 °C and then with Alexa Fluor 546-labeled secondary antibody (Invitrogen, USA) at 1:200 dilution for 2 h at room temperature. The samples were mounted and photographed under a fluorescence microscope (Carl Zeiss, Germany).

### Cell proliferation assay

rDPCs (3 × 10^3^ cells/well) were cultured in 96-well plates for 24 h and then cultured in αMEM or rDFSC-CM with or without LPS (0.5 mg/L) for 1, 2, and 3 days. The proliferation of the cells was evaluated using the Cell Counting Kit-8 assay (CCK-8, Beyotime, China). The absorbance at a wavelength of 450 nm was determined.

### Assessment of the cell cycle and apoptosis by flow cytometry

rDPCs were cultured in αMEM or rDFSC-CM with or without 0.5 mg/L LPS for 24 h, harvested by trypsinization, and fixed with 70% cold ethanol for 1 h on ice. After washing, the cells were incubated with Annexin V/7-aminoactinomycin D (7-AAD, BD Biosciences, USA) for 30 min at 4 °C in the dark. Cell apoptosis and the cell cycle distribution were analyzed using a FACSCalibur flow cytometer (BD Biosciences, USA) and Cell Quest version 3.1 software (BD Biosciences, USA) according to the manufacturer’s instructions.

### Migration assay

Wound-healing and Transwell migration assays were employed to assess the migration capacity of rDPCs after αMEM or rDFSC-CM treatment. For the wound-healing assay, rDPCs (2 × 10^5^ cells/well) were seeded in 24-well plates in αMEM with 10% FBS for 24 h to obtain a monolayer. After the cells reached 90% confluence, a 4-μm-wide wound was made with a sterile pipette tip on the monolayer. The culture was replenished with αMEM or rDFSC-CM with or without 0.5 mg/L LPS. The cells were incubated at 37 °C in an atmosphere with 5% CO_2_ for 24 h. The migrated cells were captured under an inverted light microscope (Carl Zeiss, Germany). The Transwell migration assay was performed using Transwell chambers and inserts with 8-μm pores (Corning, USA). Suspended cells (at a density of 2 × 10^5^ cells/mL in 100 μL of FBS-free medium) were seeded into the upper chambers. The lower chambers contained αMEM or rDFSC-CM with or without 0.5 mg/L LPS. After 24 h of incubation at 37 °C and 5% CO_2_, the cells were fixed with 4% paraformaldehyde (Beyotime, China) for 15 min and stained with a 1% crystal violet solution (Beyotime, China) for 30 min. The nonmigrated cells on the upper membrane were removed using cotton swabs. The cells that had migrated through the membrane were captured and counted under an inverted light microscope (Carl Zeiss, Germany).

### Mineralization assay

A total of 50,000 rDPCs were seeded into each well of six-well plates containing αMEM with 10% FBS and incubated for 24 h. The culture medium was then changed to odontogenesis-inducing medium containing 0.5 mg/L LPS with or without rDFSC-CM. For Alizarin Red staining, the cells were fixed at day 7, stained with 1% Alizarin Red S (Sigma-Aldrich, USA) at room temperature for 1 h, and washed twice with PBS. The mineralized nodules were observed and photographed under a stereomicroscope (Olympus, Japan). The expression of the odontogenic genes *alkaline phosphatase* (*ALP*), *dentin sialophosphoprotein* (*DSPP*), *Runt-related transcription factor 2* (*Runx2*), *bone sialoprotein* (*BSP*), and *dentin matrix acidic phosphoprotein 1* (*DMP1*) was evaluated by real-time quantitative PCR using the primer sequences listed in Table 2.

**Table 2:** Primers for odontogenic differentiation markers used in the real-time quantitative PCR assay

Gene	Primer sequence	
*ALP*	Forward	AACGTGGCCAAGAACATCATCA
	Reverse	TGTCCATCTCCAGCCGTGTC
*DSPP*	Forward	GGTGCTCATCTCCCATTGTGA
	Reverse	CAGGTAGCAGCGTGTGAAAGC
*Runx2*	Forward	CAGTAGCAAACCGAAACAC
	Reverse	CACACACAGACAATAAATAGCA
*BSP*	Forward	TGTGGAATGGTGCTACGGTCTC
	Reverse	GATCAACAGCCCTGATTTACGATG
*DMP1*	Forward	GAGCGATCGAGGCCATACC
	Reverse	CTCGTGATCCCCTTTAGATTCTTC
*β-Actin*	Forward	CCTCTTTGCATGTCTCACTC
	Reverse	AATGTCACGCACGATTTCC

### Detection of dentin collagen fiber synthesis in vivo

To detect the effects of rDFSC-CM on the odontogenic differentiation of rDPCs in vivo, cells (1 × 10^6^ cells/mL) were loaded onto hydroxyapatite/tricalcium phosphate scaffolds (HA/TCP, Engineering Research Centre in Biomaterials, Sichuan University, China), which were assigned to four experimental groups: bare scaffold, αMEM-treated cell-loaded scaffold, αMEM/LPS-treated cell-loaded scaffold, and rDFSC-CM/LPS-treated cell-loaded scaffold. The samples were transplanted subcutaneously into the backs of nude mice obtained from the Laboratory Animal Center at Sun Yat-sen University. The protocol was approved by the University Ethics Committee of the ZSSOM on Laboratory Animal Care (No. 2017-194). The mice were sacrificed at 8 weeks postimplantation. The samples were separated, fixed in 4% paraformaldehyde (Beyotime, China) solution for 24 h, and decalcified in commercial demineralization liquid (Beyotime, China) for 2 weeks. The samples were subsequently embedded in paraffin and cut into 5-μm-thick sections. Masson’s staining was applied to assess the dentin collagen fibers according to our previously published protocols [17]. The images were captured using an inverted light microscope. The expression of the above-described odontogenic proteins was evaluated by Western blotting. Primary antibodies against the following proteins were used: ALP (Abcam, USA; 1:1000 dilution), Runx2 (Abcam, USA; 1:1000 dilution), DSPP (Santa Cruz, USA; 1:2000 dilution), and vinculin (Abcam, USA; 1:5000 dilution). The densities of the protein bands were quantified using ImageJ version 1.50i software (Bethesda, USA).

### Rat pulpitis induction and rDFSC-CM administration

Eight-week-old male S-D rats weighing 240–280 g (*N* = 30) were purchased from the Laboratory Animal Center at Sun Yat-sen University. The experiments were approved by the University Ethics Committee of the ZSSOM on Laboratory Animal Care (No. 2017-195). After the induction of anesthesia through the intraperitoneal injection of 10% chloral hydrate, the rats underwent drilling of the maxillary first molar on the occlusal surface using a high-speed handpiece and a round diamond bur (Shofu, Canada). Coronal pupal exposure was then confirmed using a sterile size 10 K-file that was sequentially increased to a size 40 K-file. After hemostasis was achieved, the injured site was directly capped with an aseptic gelatin sponge soaked in rDFSC-CM or αMEM with 10 mg/mL LPS and then sealed with light-cured glass ionomer cement (3M, USA). Twenty-four hours later, half of the animals were sacrificed, and their teeth were extracted, fixed, decalcified, embedded in paraffin, and cut into 5-μm-thick sections as described above. Hematoxylin-eosin (HE) staining was used for evaluation of inflammatory infiltration. The rest of the rats were sacrificed 7 days later, and their teeth were carefully extracted for immunohistochemistry with anti-Runx2 (1:200, Abcam, USA). Images were captured with an inverted light microscope.

### Statistical analysis

The data are presented as the means ± standard deviations (SDs) from at least three independent experiments and were analyzed using GraphPad Prism version 6.0 software (GraphPad, Inc., USA). Student’s *t* test was selected for pairwise comparisons, and one-way analysis of variance (ANOVA) was used for multiple comparisons. Differences with *P* < 0.05 were considered statistically significant.

## Results

### Identification of rDFSCs and rDPCs

**Fig. 1:**
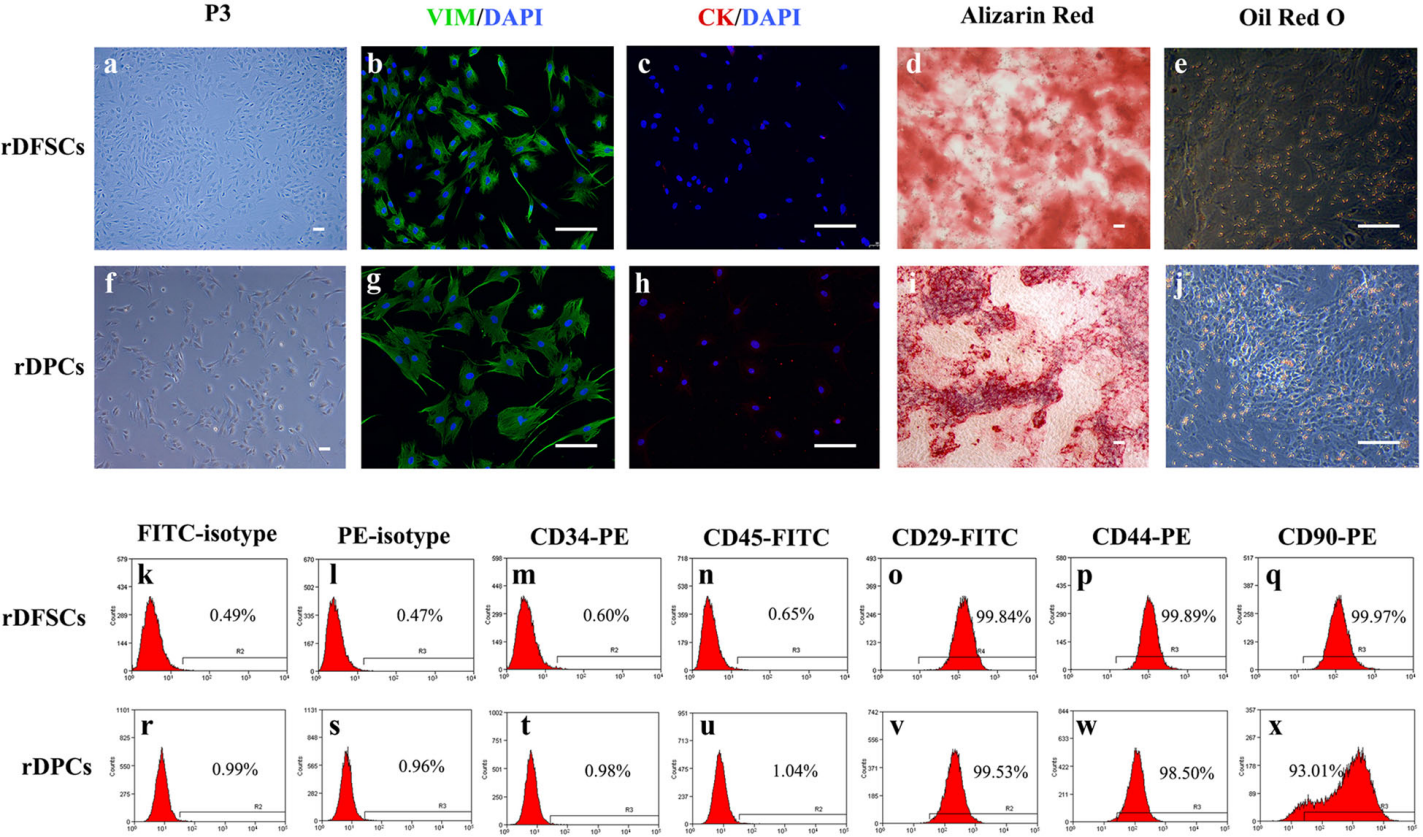
Isolation and identification of rDFSCs and rDPCs. Representative images of rDFSCs (**a**) and rDPCs (**f**). rDFSCs and rDPCs were both positive for vimentin (**b**, **g**) and negative for cytokeratin (**c**, **h**). After osteogenic induction for 14 days, mineralized nodules were detected in rDFSCs (**d**) and rDPCs (**i**) by Alizarin Red staining. After adipogenic induction for 14 days, lipid droplets were detected in rDFSCs (**e**) and rDPCs (**j**) by Oil Red O staining. Scale bar 100 μm. Representative flow cytometric results showed the expression pattern of CD markers in P3 rDFSCs (**k**–**q**) and rDPCs (**r**–**x**)

DFSCs and rDPCs were successfully isolated from rat dental follicle or dental pulp tissues (Fig. 1a, f), and immunofluorescence analysis showed that rDFSCs and rDPCs were both positive for the MSC marker vimentin (Fig. 1b, g) and negative for the epithelial marker cytokeratin (Fig. 1c, h). A flow cytometric analysis showed that the rDFSCs expressed high levels of the MSC markers CD29 (99.84%), CD44 (99.89%), and CD90 (99.97%) and were negative for the hematopoietic markers CD34 (0.60%) and CD45 (0.65%) (Fig. 1k–q). rDPCs also expressed high levels of the MSC markers CD29 (99.53%), CD44 (98.50%), and CD90 (93.01%) and were negative for CD34 (0.98%) and CD45 (1.04%) (Fig. 1r–x). In addition, after induction in osteogenic medium for 14 days, both rDFSCs and rDPCs formed mineralized nodules that were visualized by Alizarin Red staining (Fig. 1d, i). After culture in adipogenic medium for 14 days, both rDFSCs and rDPCs formed lipid droplets, as visualized by Oil Red O staining (Fig. 1e, j). These results demonstrated the stem cell characteristics and multipotent differentiation abilities of rDFSCs and rDPCs.

### rDFSC-CM attenuated inflammation in LPS-stimulated rDPCs

**Fig. 2:**
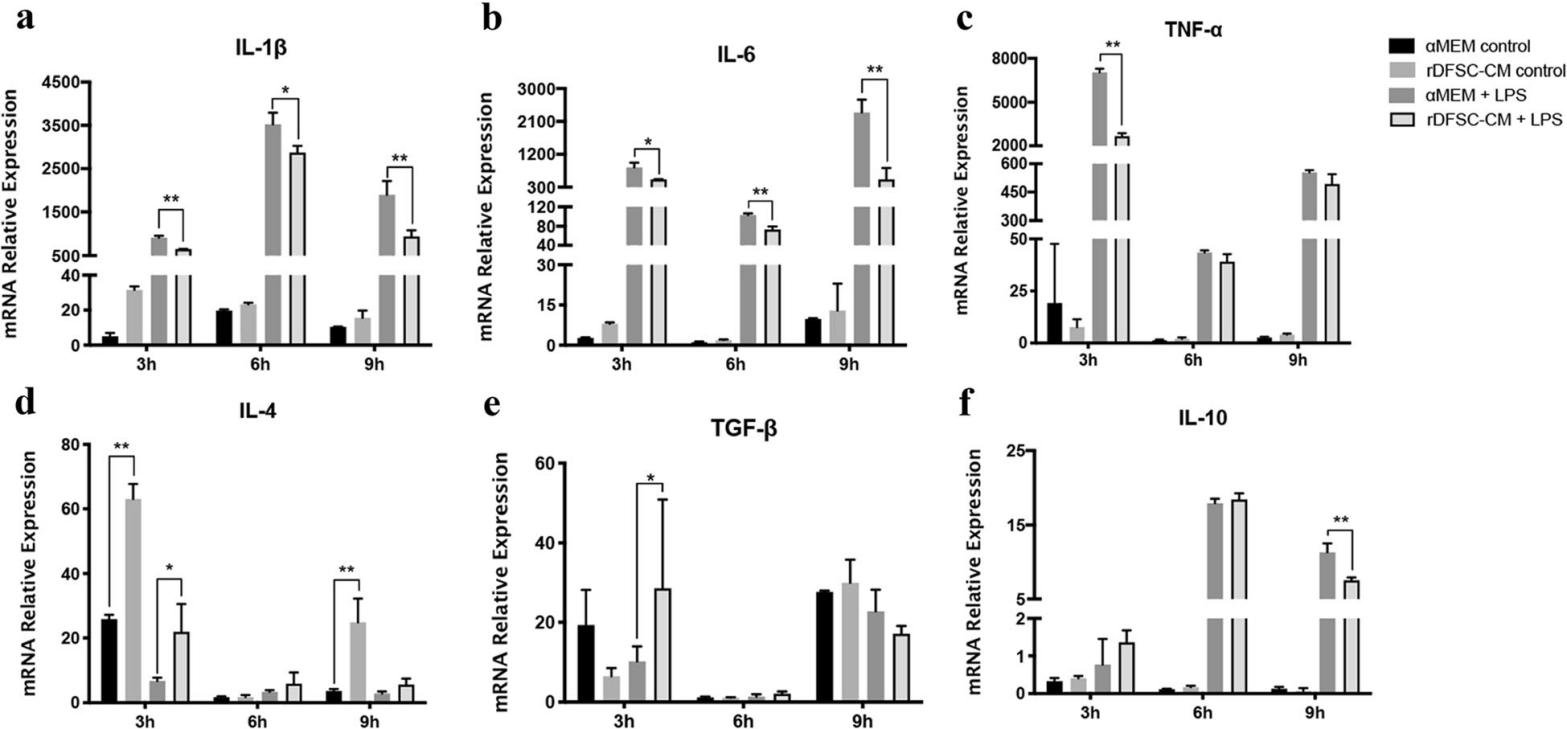
rDFSC-CM attenuated inflammation in LPS-stimulated rDPCs. Cells were cultured in rDFSC-CM or αMEM with or without 0.5 mg/L LPS for 3, 6, and 9 h. As demonstrated by real-time PCR, rDFSC-CM treatment decreased the expression of IL-1β (**a**), IL-6 (**b**), and TNF-α (**c**); elevated the expression of IL-4 (**d**) and TGF-β (**e**); and downregulated the level of IL-10 (**f**). β-Actin was used as an internal control. The data are presented as the means ± SDs from three independent experiments. *P < 0.05, **P < 0.01

The mRNA levels of the proinflammatory cytokines IL-1β, IL-6, and TNF-α were significantly higher in rDPCs exposed to 0.5 mg/L LPS for 3, 6, and 9 h than in the control group. However, treatment with rDFSC-CM downregulated the expression of proinflammatory cytokines in LPS-stimulated rDPCs (Fig. 2a–c). Furthermore, treatment with rDFSC-CM for 3 h promoted the expression of the anti-inflammatory cytokines IL-4 and TGF-β in LPS-stimulated rDPCs, although the level of IL-10 was downregulated after 9 h of treatment (Fig. 2d–f). These results demonstrated that rDFSC-CM could ameliorate LPS-induced inflammation in rDPCs.

### The ERK1/2 and NF-κB signaling pathways were involved in rDFSC-CM-mediated anti-inflammatory effects on LPS-stimulated rDPCs

**Fig. 3:**
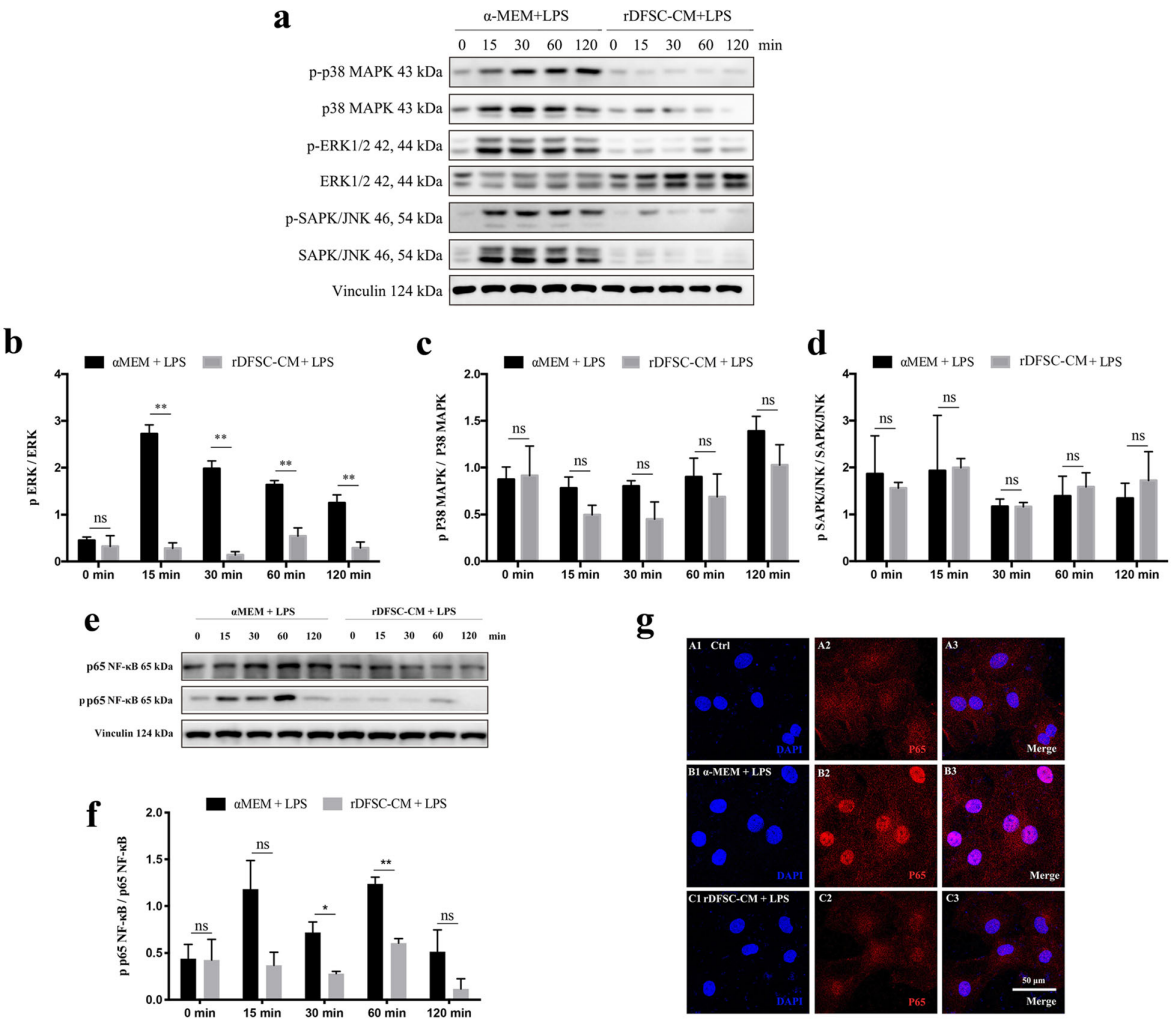
Effects of rDFSC-CM on MAPK and NF-κB signaling in inflammatory rDPCs. Cells were cultured until reaching 80% confluence, the medium was then changed to rDFSC-CM or αMEM containing 0.5 mg/L LPS, and the cells were then incubated for 0, 15, 30, 60, and 120 min. The activation of the MAPK and NF-κB signaling pathways was detected by a Western blot analysis of total cellular proteins and immunofluorescence staining. **a** Phosphorylation levels of ERK 1/2, p38 MAPK, and SAPK/JNK. Representative photographs of immunoblots are shown. The molecular weights of the bands are indicated in kilodaltons. **b**–**d** Relative densitometry analysis of the aforementioned proteins showed that rDFSC-CM significantly suppressed the phosphorylation of ERK1/2 but not that of p38 or SAPK/JNK. **e** Phosphorylation level of NF-κB p65. **f** A relative densitometry analysis of NF-κB p65 showed that p65 phosphorylation increased and peaked at 60 min after LPS treatment but was decreased by rDFSC-CM treatment. **g** The subcellular localization of NF-κB p65 after treatment with rDFSC-CM for 60 min was determined by immunofluorescence staining with Alexa Fluor 546-conjugated secondary antibody. DAPI was used for DNA staining. Representative images of three independent experiments are shown (scale bar 50 μm). The data are presented as the means ± SDs from at least three independent experiments. **P < 0.01; ns, no significant difference

Stimulation with LPS for 15 to 120 min induced the phosphorylation of ERK1/2 in rDPCs in a time-dependent manner, and ERK1/2 phosphorylation was significantly suppressed by rDFSC-CM treatment (Fig. 3a, b). In contrast, the phosphorylation of P38 or SAPK/JNK induced by either LPS stimulation or rDFSC-CM treatment differed little (Fig. 3a, c, d). The phosphorylation of p65 was increased and peaked at 60 min after LPS treatment but decreased after rDFSC-CM treatment (Fig. 3e, f). This finding was further confirmed by immunofluorescent staining, which showed that the expression of nuclear p65 was increased by LPS stimulation but reduced by rDFSC-CM treatment for 60 min (Fig. 3g).

### Effects of rDFSC-CM on the proliferation, apoptosis, cell cycle, and migration of LPS-stimulated rDPCs

**Fig. 4:**
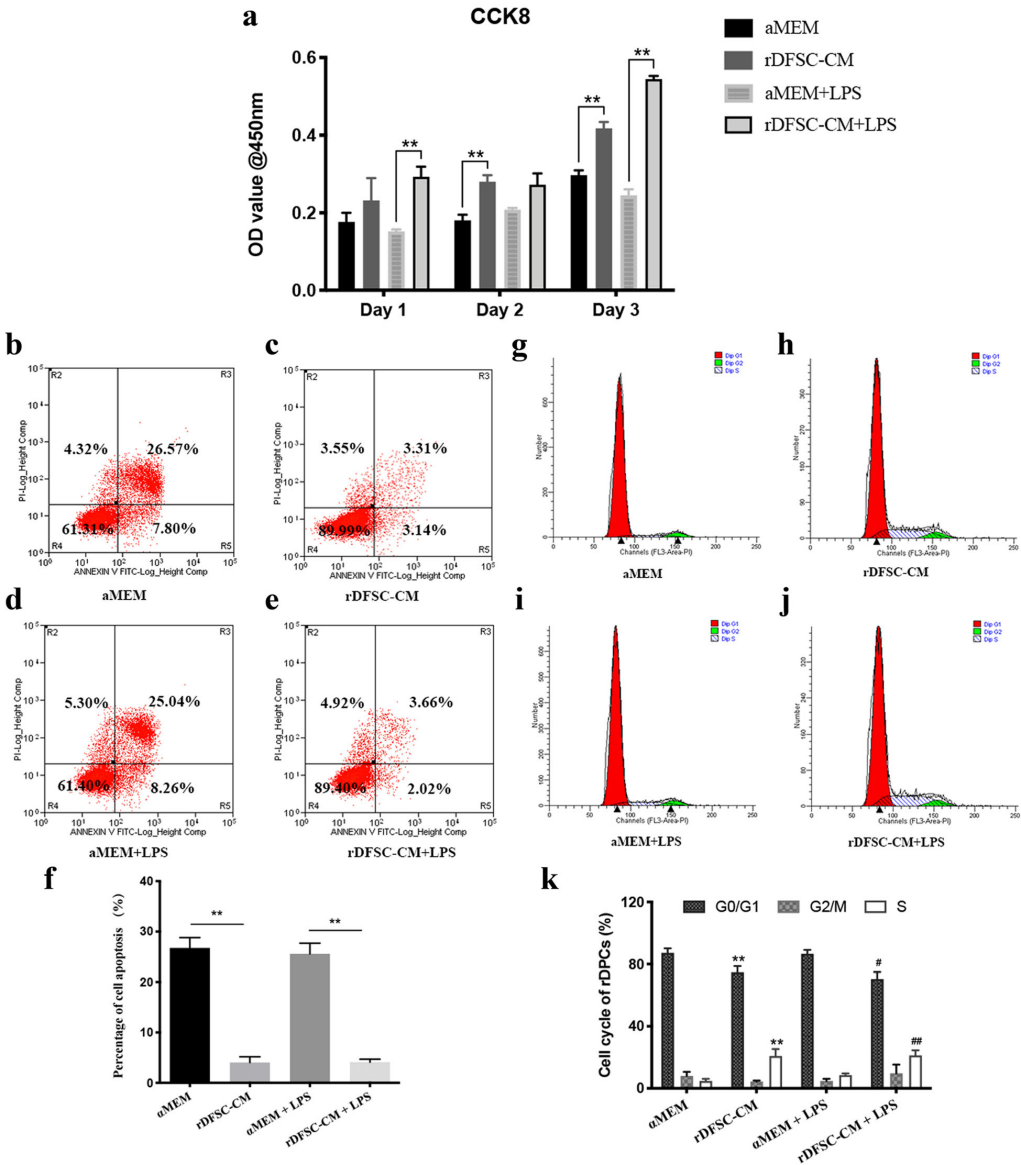
Proliferation, apoptosis, and cell cycle of inflammatory rDPCs after treatment with rDFSC-CM. Cells were cultured until reaching 80% confluence, and the medium was then changed to rDFSC-CM or αMEM with or without 0.5 mg/L LPS. **a** The proliferation of rDPCs after rDFSC-CM treatment for 1, 2, and 3 days was determined using the Cell Counting Kit-8 assay. The results are presented as the means ± SDs from at least three independent experiments. **P < 0.01. **b**–**e** The apoptosis of rDPCs was downregulated by rDFSC-CM treatment for 24 h, as detected by flow cytometry. Representative images of three independent experiments are shown. **f** Statistical analysis of cell apoptosis. The results are presented as the means ± SDs. **P < 0.01. **g**–**j** The cell cycle distribution of rDPC treatment with rDFSC-CM for 24 h was determined by flow cytometry. Representative images of three independent experiments are shown. **k** Statistical analysis of the cell cycle. The results are presented as the means ± SDs. *Compared with the αMEM group, ^#^Compared with the αMEM + LPS group, **P < 0.01, ^#^*P* < 0.05, ^##^*P* < 0.01

**Fig. 5:**
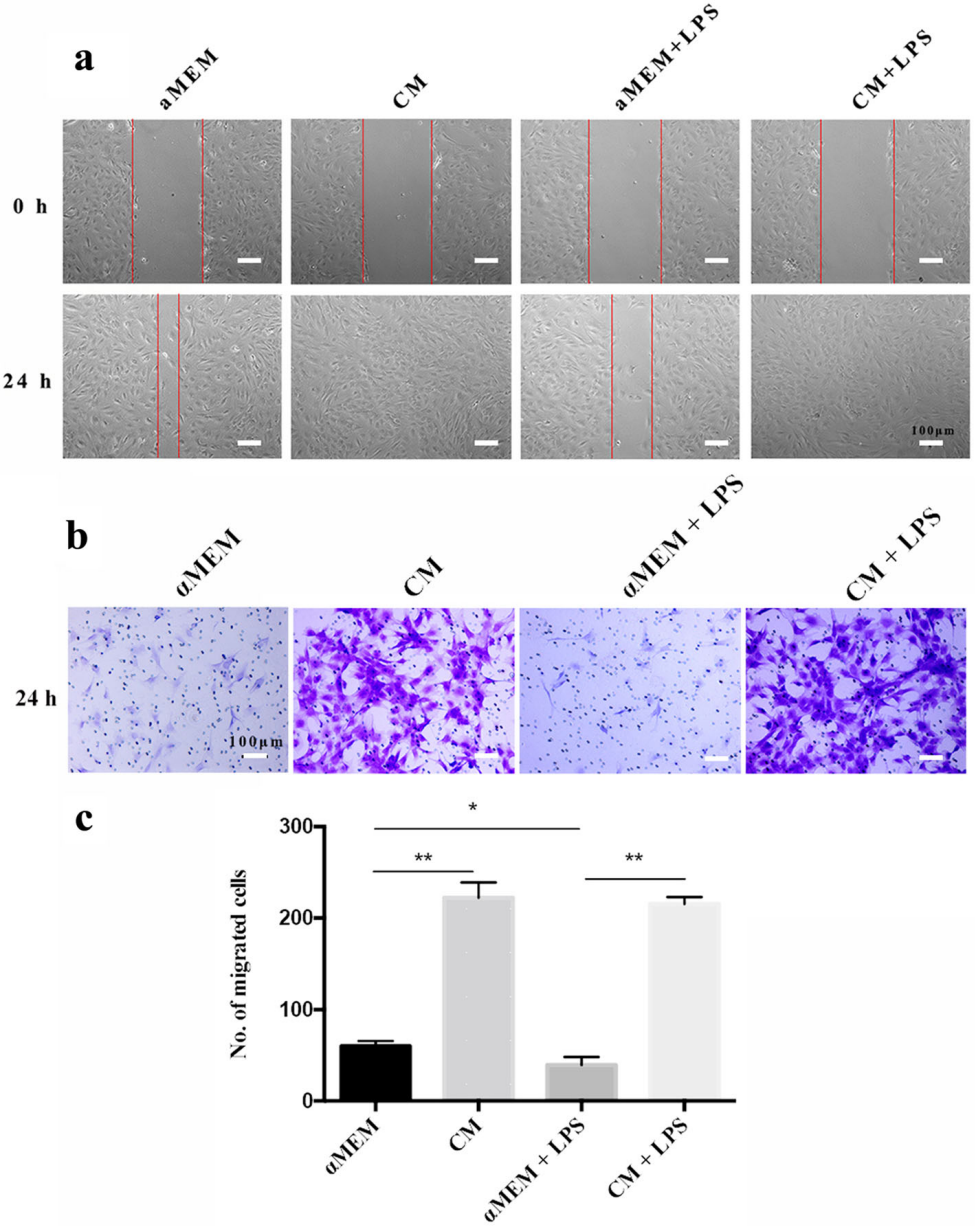
Migration capacity of inflammatory rDPCs after treatment with rDFSC-CM for 24 h. **a** Results of the wound-healing assay. Representative images of three independent experiments are shown (scale bar 100 μm). **b** Microscopic view of migrated cells stained with crystal violet solution. Representative images of three independent experiments are shown (scale bar 100 μm). **c** Statistical analysis of the number of migrated cells. The results are presented as the means ± SDs. *P < 0.05, **P < 0.01

As shown in Fig. 4a, rDFSC-CM treatment for 1 to 3 days significantly increased the proliferation of rDPCs, and treatment for 24 h downregulated the apoptosis of these cells (Fig. 4b–f). Furthermore, rDFSC-CM treatment for 24 h significantly decreased the proportion of rDPCs at the G0/G1 phase and increased that at S phase (Fig. 4g–k). Cell migration was decreased by LPS stimulation for 24 h, but significantly increased by rDFSC-CM treatment (Fig. 5a–c).

### rDFSC-CM facilitated the odontogenic differentiation and ectopic dentinogenesis of inflammatory rDPCs

**Fig. 6:**
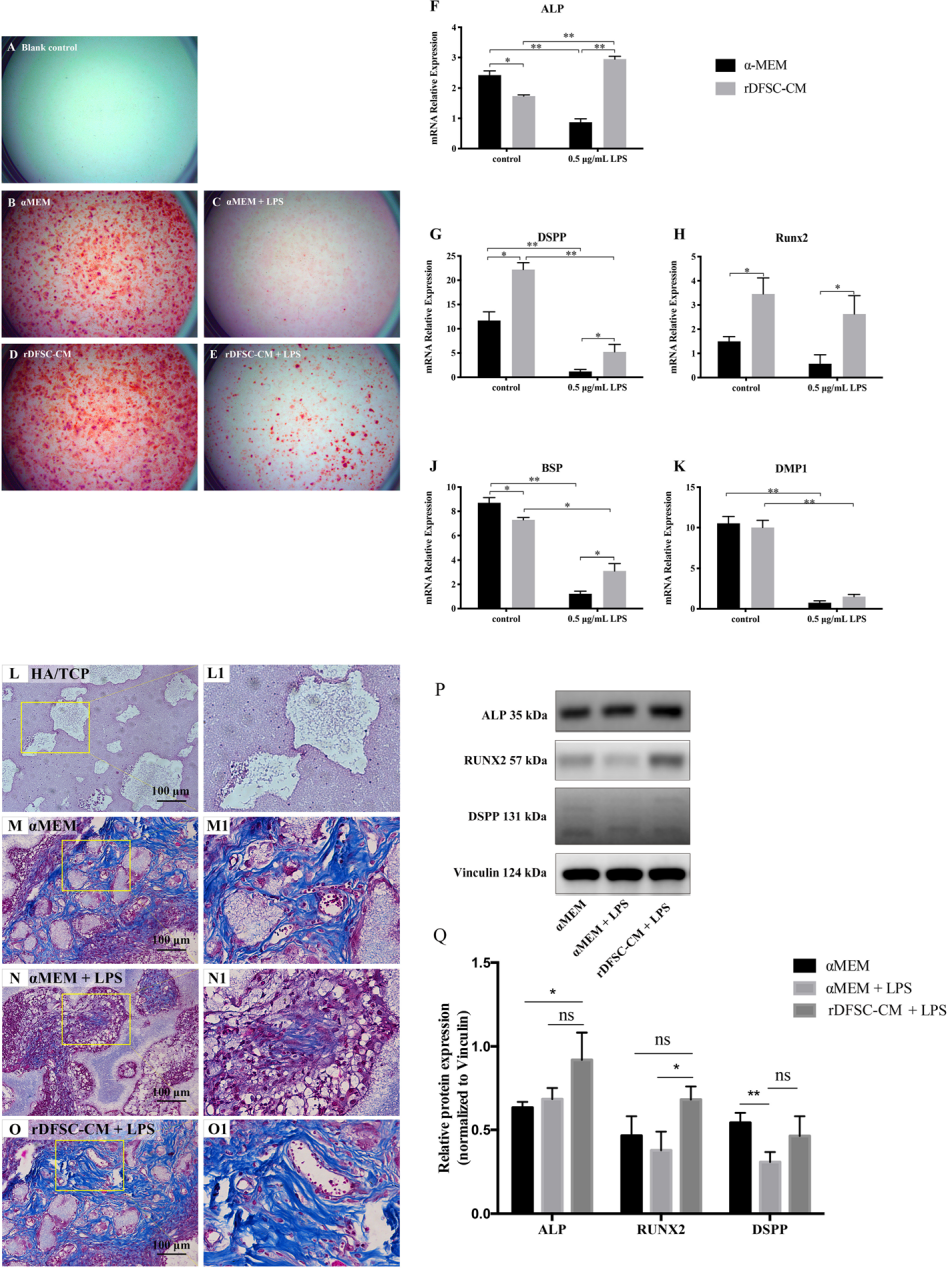
rDFSC-CM promoted the odontogenic differentiation and ectopic dentinogenesis of rDPCs*.***A**–**E** Mineralized nodule formation of inflammatory rDPCs after odontogenic induction for 7 days. **A** Blank control group. **B** αMEM group. **C** αMEM + LPS group. **D** rDFSC-CM group. **E** rDFSC-CM + LPS group. Representative images of three independent experiments are shown. **F**–**K** Real-time PCR revealed that the treatment of inflammatory rDPCs with rDFSC-CM for 7 days upregulated the expression of the odontogenic genes *ALP*, *DSPP*, *Runx2*, *BSP*, and *DMP1*. β-Actin was used as an internal control. **L**–**O** Effects of rDFSC-CM on ectopic dentin collagen fibers of inflammatory rDPCs in vivo. The cells were loaded onto HA/TCP and then transplanted into the subcutaneous tissue of nude mice, where they remained for 8 weeks. The Masson staining results showed the production of collagen fibers (× 20). **L**, **L1** Blank control group. **M**, **M1** αMEM group. **N**, **N1** αMEM + LPS group. **O**, **O1** rDFSC-CM + LPS group. Representative images of three independent experiments are shown (scale bar 100 μm). **P**, **Q** As demonstrated by Western blot analysis, treatment with rDFSC-CM for 8 weeks significantly increased the expression of Runx2 in inflammatory rDPCs after in vivo. The levels of the ALP and DSPP proteins were also increased in the rDFSC-CM group, although the differences were not statistically significant. The molecular weights of the bands are indicated in kilodaltons. Vinculin was used as an internal control. The results are presented as the means ± SDs from at least three independent experiments. *P < 0.05, **P < 0.01; ns, no significant difference

To explore the impact of rDFSC-CM on the odontogenic differentiation of rDPCs under inflammatory conditions, the cells were cultured in LPS-containing odontogenic medium with or without rDFSC-CM. After 7 days of induction, markedly improved mineralized matrix deposition was observed after rDFSC-CM treatment compared with αMEM treatment, as demonstrated by Alizarin Red S staining (Fig. 6A–E). Real-time PCR revealed that the expression of the odontogenesis-related genes *ALP*, *DSPP*, *Runx2*, *BSP*, and *DMP1* was increased by rDFSC-CM treatment compared with αMEM treatment (Fig. 6F–K).

We subsequently evaluated the effects of rDFSC-CM on the ectopic dentinogenesis of rDPCs in vivo. HA/TCP scaffolds were used to load rDFSC-CM-treated rDPCs, and the scaffolds were implanted subcutaneously into the backs of nude mice and maintained in the mice for 8 weeks. As demonstrated by Masson’s staining, the production of collagen fibers in rDPCs was decreased under inflammatory conditions but increased by rDFSC-CM treatment (Fig. 6L–O). A Western blot analysis also revealed a significant increase in Runx2 expression in inflammatory rDPCs treated with rDFSC-CM. Furthermore, the ALP and DSPP protein levels were also increased in the rDFSC-CM group, although the differences were not statistically significant (Fig. 6P, Q).

### rDFSC-CM suppressed inflammatory infiltration and promoted Runx2 expression in rat pulpitis tissue

**Fig. 7:**
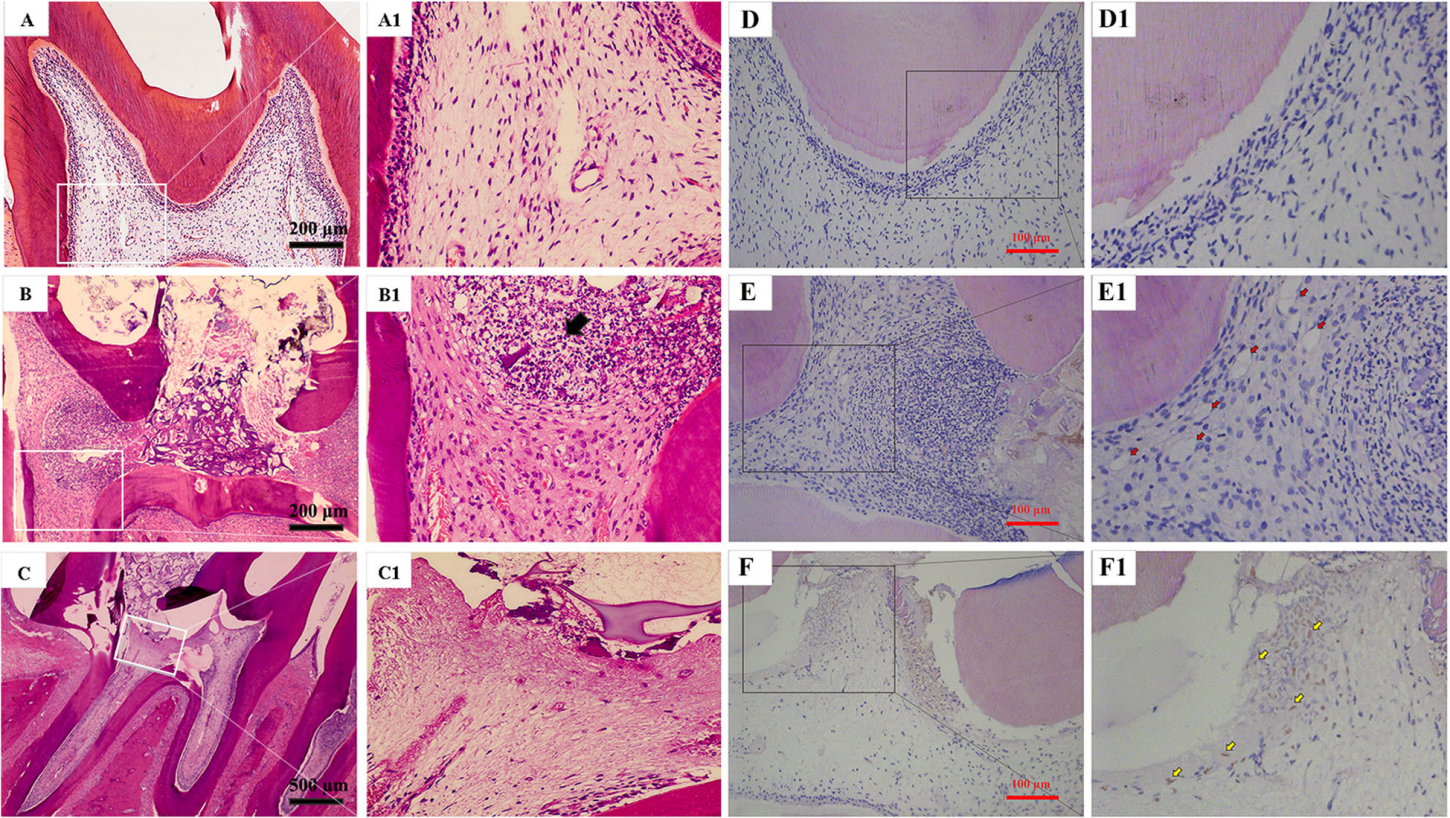
Inflammatory infiltration and Runx2 expression in an experimental pulpitis model of rats after direct pulp capping with rDFSC-CM. **A**–**C** The degree of inflammatory infiltration in rats with experimental pulpitis in which the pulp was directly capped with rDFSC-CM for 24 h was determined by HE staining. **A**, **A1** Blank control group (× 10). **B**, **B1** The infiltration of inflammatory cell in the αMEM + LPS group is indicated by a black arrow (× 10). **C**, **C1** rDFSC-CM + LPS group (× 4). Representative images of three independent experiments are shown. **D**–**F** The expression of Runx2 in an experimental pulpitis model in rats after direct pulp capping with rDFSC-CM for 7 days was revealed by immunofluorescence staining (× 20). **D**, **D1** Blank control group. **E**, **E1** αMEM + LPS group. Capillary dilatation is indicated by red arrows. **F**, **F1** rDFSC-CM + LPS group. Some of the cells were positive for Runx2 (yellow arrows). Representative images of three independent experiments are shown

To study whether rDFSC-CM can reduce inflammatory infiltration and promote the repair of dental pulp tissue after injury, an experimental pulpitis model was constructed in rats by using 10 mg/mL LPS. HE staining revealed that 24 h of treatment resulted in typical inflammation, as evidenced by the infiltration of a large number of inflammatory cells (black arrows) in the coronal pulp and the dilatation of blood vessels in the radicular pulp, which indicated the successful induction of pulpitis (Fig. 7A, B). Intriguingly, slight inflammatory infiltration or capillary dilatation was observed in the pulp capped with rDFSC-CM for 24 h (Fig. 7C). After 7 days of treatment with LPS, capillary dilatation was still observed (red arrows). A large number of inflammatory cells had infiltrated and were distributed diffusely, and few Runx2-positive cells observed (Fig. 7D, E). In contrast, rDFSC-CM treatment suppressed inflammatory cell infiltration and triggered Runx2 expression (yellow arrows) in some of the odontoblast-like cells near the injured site (Fig. 7F).

## Discussion

DPCs, the most abundant cell types in dental pulp, are the main cells that function in host defenses and tissue regeneration in the pulp-dentin complex [18]. When exposed to bacteria and/or their toxic components, DPCs can recognize invading bacteria via their expression of a variety of pattern recognition receptors [1, 19–21] and induce an innate immune response by expressing antimicrobial peptides (AMPs) and secreting various inflammatory cytokines, such as TNF-α, IL-6, IL-8, IL-1α, CCL7, CCL26, and CXCL11 [19, 22–26], and these effects lead to the recruitment of macrophages, neutrophils, and other immune cells and initiation of the early inflammatory response to eliminate invading microbes [1]. Moreover, DPCs subsequently migrate to the injured site, where they differentiate into odontoblast-like cells to synthesize reparative dentin, initiating repair and regeneration [27–30]. However, antibacterial activities easily induce the inflammatory cascade and thereby propagate sustained inflammation [2–4], which not only irreversibly damages vital pulp tissue but also actively impedes the repair responses of DPCs, and these effects eventually lead to total necrosis of the pulp [1, 24]. Hence, attenuation of inflammation is a prerequisite for the initiation of pulp healing and regeneration. The vital importance of DPCs in the dynamic interplay between inflammation and regeneration should be brought to the forefront.

DFSCs, which can be easily obtained from the dental follicle of impacted third molars, exhibit enhanced proliferation and differentiation capacities and exert a stronger immunomodulatory effect on lymphocytes and Treg cells than other dental MSC types, such as stem cells from human exfoliated deciduous teeth (SHEDs) and DPSCs [31, 32]. Our previous study demonstrated that rDFSC-CM plays an immunomodulatory role in rat acute lung injury by suppressing the levels of proinflammatory cytokines and reprogramming of macrophages toward the anti-inflammatory M2 phenotype [16]. During tooth development, the dental follicle encompasses the dental papilla (the precursor of dental pulp), and cells from both different tissues intimately involved, which allows the creation of specific local niches for tissue development. We found that the indirect coculture of DFSCs and dental papilla cells activates the expression of reprogramming markers and inhibits the apoptosis of dental papilla cells [33], which suggests that DFSC-CM optimizes the extracellular microenvironment to enhance the reprogramming capacity of other dental cells. Accordingly, we hypothesize that rDFSC-CM is a prospective option for ameliorating the inflammatory state of the injured dental pulp and promoting pulp regeneration due to its immunomodulatory effects on rDPCs.

To verify this hypothesis, we first investigated the immunomodulatory effects of rDFSC-CM on inflammatory rDPCs. Consistent with a previous study [34], rDPCs showed a rapid response to LPS exposure and presented upregulated gene expression of IL-1β, IL-6, and TNF-α. rDFSC-CM treatment significantly downregulated the levels of these proinflammatory cytokines and increased the mRNA levels of the anti-inflammatory cytokines IL-4 and TGF-β in inflammatory rDPCs. In addition, rDFSC-CM significantly inhibited the phosphorylation of ERK1/2 and p65 and suppressed the nuclear translocation of p65, which indicated that rDFSC-CM regulated the expression of proinflammatory and anti-inflammatory cytokines in rDPCs by inhibiting activation of the inflammatory MAPK and NF-кB signaling pathways. To further detect whether rDFSC-CM can alleviate pulp inflammation in vivo, an experimental pulpitis model was generated in rats, and the model rats presented the diffuse infiltration of inflammatory cells in the pulp after 7 days of LPS treatment. When the pulp was capped with rDFSC-CM, however, the degree of pulpal inflammation subsided substantially, and only slight inflammatory cell infiltration or capillary dilatation observed. These in vitro and in vivo results indicated the immunomodulatory capacity of rDFSC-CM in alleviating the inflammatory responses of DPCs and injured dental pulp.

Injured dental pulp has an innate capacity for self-regeneration. However, sustained inflammation might cause an imbalance in the dynamic equilibrium of the dental pulp and suppress pulp regeneration mediated by DPCs [24]. In the present study, the reduced expression of proinflammatory cytokines in inflammatory rDPCs treated with rDFSC-CM was accompanied by increased cell proliferation and transformation from the G1/G0 to the S phase, which indicated that rDFSC-CM can expedite the cell cycle and thus accelerate cell proliferation. In addition, treatment with rDFSC-CM significantly promoted the migration of inflammatory rDPCs but decreased cell apoptosis. These data indicated that the regenerative capacities of inflammatory rDPCs were improved. On the other hand, rDFSC-CM enhanced mineralized nodule formation and odontogenic gene expression in vitro and the ectopic dentin collagen fiber deposition of inflammatory rDPCs in vivo, and these findings indicate that rDFSC-CM exerts advantageous effects on the odontoblastic differentiation of inflammatory rDPCs via immunomodulatory pathways. A rat model of direct pulp capping provided further evidence of the repair and regeneration cascade in the inflamed pulp. rDFSC-CM treatment inhibited the infiltration of inflammatory cells and triggered the expression of the mineralized marker Runx2 in some of the odontoblast-like cells in proximity to the injured site. Taken together, these results demonstrate that rDFSC-CM might help create an optimal microenvironment that favors tissue repair and regeneration due to its immunomodulatory effects on primary immune cells in the injured pulp, including rDPCs.

In our previous study, we characterized the soluble factors in rDFSC-CM by protein array analysis and found that rDFSC-CM contains 42 proteins at levels that were at least 2-fold greater than those in the control group [16]. To identify the possible effective components, we performed a cluster analysis of these proteins and identified 26 factors that are known to exhibit functional properties that might be beneficial for the treatment of pulpitis (Table 3). TGF-β3 and TSP-1 can reprogram macrophages into the anti-inflammatory M2 phenotype [16], and TGF-β3 also induces ectopic mineralization in DPCs to regulate their differentiation to odontoblasts [42]. FGF-2 effectively promotes the proliferation and odontogenic differentiation of DPCs under inflammatory conditions [61] and enhances the in vivo ectopic formation of dentin-like material of stem cells from inflamed dental pulp tissue [39]. OPN plays a nonredundant role in regulating the formation and mineralization of mouse dentin [40]. Leptin can induce angiogenesis, odontogenic differentiation, and mineralization in human DPCs [41]. VEGF promotes the in vitro angiogenesis of human DPCs through modulation of the expression of lysyl oxidase [46]. A specific distribution of NGFR was observed in the tooth buds and dental follicle [62]. NRP-2 not only functions as a receptor for semaphorins, which are a family of neural axon guidance factors, but also interacts with VEGFs [63]. Considering the multiple functions and interactions of the identified factors, we hypothesize that the combinatorial effects of these factors in rDFSC-CM might provide therapeutic benefits for the treatment of pulpitis.

**Table 3:** Differentially expressed (> 2-folds) soluble factors in rDFSC-CM compared with serum-free control medium identified by a protein array

	Ratio	References
**Anti-inflammation**		
TSP-1	105.9	[35]
ACTH	96.5	[36]
TRAIL	23.0	[37]
AMPK-α1	10.4	[38]
FGF-2	3.1	[39]
**Odonto/osteogenesis**		
OPN	69.5	[40]
Leptin	68.1	[41]
TGF-β3	18.6	[42]
EGFR	8.6	[43]
MMP-2	4.2	[44]
β-Catenin	3.3	[45]
**Angiogenesis**		
VEGF	38.4	[46]
VEGF-C	24.4	[47]
FGF-BP	15.5	[48]
FSL-1	11.9	[49]
TIE-2	8.2	[50]
**Neuroprotection**		
NGFR	640.5	[51]
NRP-2	74.5	[52]
Orexin A	42.7	[53]
MUSK	24.4	[54]
GFR-α2	6.9	[55]
BDNF	4.6	[56]
**Pro-proliferation/anti-apoptosis**		
TIMP-1	87.5	[57]
TIMP-2	3.1	[58]
Ubiquitin	17.9	[59]
CXCR-4	5.4	[60]

Over the past decades, root canal treatment, which entails the removal of dental hard tissue and the entire pulp to eliminate intracanal infection, has been the therapy of choice for mature teeth with pulpitis. Inherent in this procedure is the loss of physiological and defensive functions of the pulp and the subsequent weakening of the treated teeth, which makes them more susceptible to fracture [64]. Therefore, the preservation of pulp with sustained vitality and the development of minimally invasive biological therapies have become key themes in contemporary regenerative endodontics [65]. Vital pulp therapy (VPT), in which the inflamed tissue is selectively removed and the exposed pulp is capped with bioactive materials [66, 67], provides an option to maintain the vitality of dental pulp under inflammatory conditions. Unfortunately, the most widely used capping materials, such as calcium hydroxide paste (Ca [OH]_2_) and mineral trioxide aggregate (MTA), are short of either anti-inflammatory or reparative properties, and the resins present in their formulations increase the cytotoxicity to pulp tissue, which limits the long-term success of VPT [68, 69]. MSC-based biotherapy serves as a new therapeutic breakthrough. Various in vivo studies have shown the critical roles of MSCs as therapeutic agents in modulating inflammatory processes and encouraging functional recovery in acute kidney injury, spinal cord injury, experimental colitis, and skin wounds through paracrine pathways [10, 70–72]. A recent study found that DFSCs, a candidate MSC type for MSC-based therapy, downregulate the Th2-mediated immune response of mononuclear cells by secreting TGF-β in patients with asthma [73]. We also showed that rDFSC-CM contains a rich cocktail of numerous soluble molecules, including immunomodulatory factors, growth factors, and neurotrophic factors [16]. The present study proves the favorable effects of rDFSC-CM in ameliorating inflammation and promoting the regeneration of the inflamed pulp and thus indicates the prospective application of rDFSC-CM in the development of novel capping agents for VPT or even in the treatment of other immune and inflammatory diseases. For successful clinical translation, protocols for DFSC isolation and ex vivo preparation will need to be optimized in future research.

## Conclusions

**Fig. 8:**
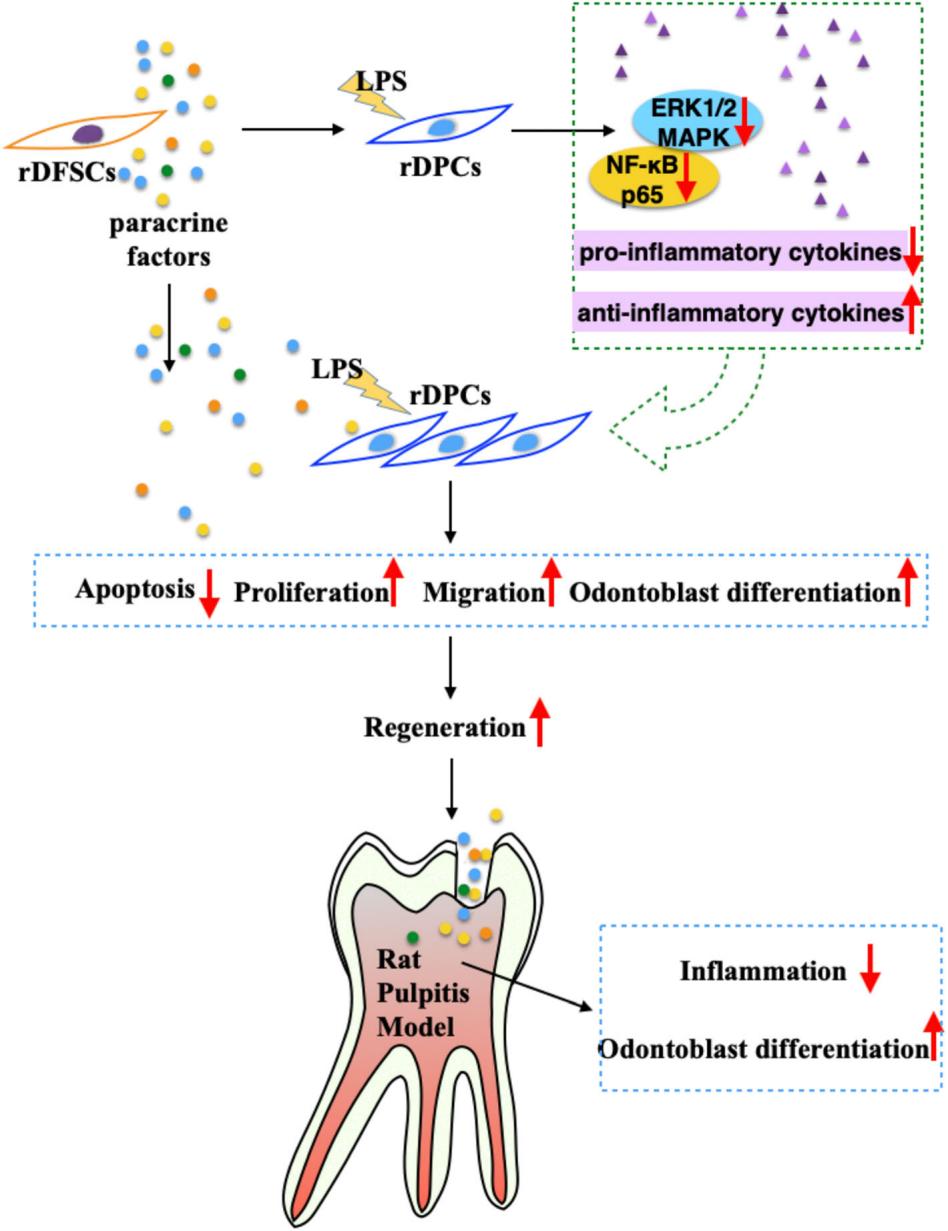
rDFSC-CM remodels the inflammatory microenvironment of injured dental pulp

In summary, the present study provides novel evidence showing that rDFSC-CM exerts its therapeutic potential on pulpitis in vitro and in vivo through immunomodulatory pathways. rDFSC-CM exhibited an optimal capability to promote the regeneration of injured pulp by remodeling a proinflammatory microenvironment toward a regenerative microenvironment (Fig. 8). These findings confirmed our hypothesis, indicated that rDFSC-CM is a prospective biotherapeutic agent, and shed new light on MSC-based therapeutic strategies for regenerative endodontics.

## Data Availability

Please contact the author for data requests.

## Abbreviations

MSC-miVPT: Mesenchymal stem cell-based minimally invasive vital pulp therapy
rDFSCs: Rat dental follicle stem cells
CM: Conditioned medium
LPS: Lipopolysaccharide
rDPCs: Rat dental pulp cells
SHEDs: Stem cells from human exfoliated deciduous teeth
AMPs: Antimicrobial peptides
IL: Interleukin
TNF-α: Tumor necrosis factor-α
TGF-β: Transforming growth factor-β
IDO: Indoleamine 2,3-dioxygenase
PGE2: Prostaglandin E2
HGF: Hepatocyte growth factor
TSP-1: Thrombospondin 1
FGF-2: Fibroblast growth factor 2
OPN: Osteopontin
VEGF: Vascular endothelial cell growth factor
NGFR: Nerve growth factor receptor
NRP-2: Neuropilin 2
MAPK: Mitogen-activated protein kinase
NF-кB: Nuclear factor-κB
ERK: Extracellular signal-regulated kinase
ALP: Alkaline phosphatase
DSPP: Dentin sialo phosphoprotein
Runx2: Runt-related transcription factor 2
BSP: Bone sialoprotein
DMP1: Dentin matrix acidic phosphoprotein 1
LTA: Lipoteichoic acid
Ca [OH]_2_: Calcium hydroxide paste
MTA: Mineral trioxide aggregate
CCK-8: Cell Counting Kit-8
S-D: Sprague-Dawley
αMEM: α-Minimum essential medium
PBS: Phosphate-buffered saline
HA/TCP: Hydroxyapatite/tricalcium phosphate
RT-qPCR: Real-time quantitative polymerase chain reaction
HE: Hematoxylin-eosin
SD: Standard deviation
ANOVA: Analysis of variance
